# Footshock-Induced Abstinence from Compulsive Methamphetamine Self-administration in Rat Model Is Accompanied by Increased Hippocampal Expression of Cannabinoid Receptors (CB1 and CB2)

**DOI:** 10.1007/s12035-021-02656-8

**Published:** 2022-01-03

**Authors:** Subramaniam Jayanthi, Ritvik Peesapati, Michael T. McCoy, Bruce Ladenheim, Jean Lud Cadet

**Affiliations:** Molecular Neuropsychiatry Research Branch, DHHS/NIH/NIDA Intramural Research Program, 251 Bayview Boulevard, Baltimore, MD 21224 USA

**Keywords:** Abstinence, Addiction, Cannabinoid receptors, Endocannabinoid, Footshocks, Hippocampus, Methamphetamine, Methamphetamine use disorder, Neuroprotection

## Abstract

Methamphetamine (METH) use disorder (MUD) is characterized by compulsive and repeated drug taking despite negative life consequences. Large intake of METH in humans and animals is accompanied by dysfunctions in learning and memory processes. The endocannabinoid system (ECS) is known to modulate synaptic plasticity and cognitive functions. In addition, the ECS has been implicated in some of the manifestations of substance use disorders (SUDs). We therefore sought to identify potential changes in the expression of various enzymes and of the receptors (CB1 and CB2) that are members of that system. Herein, we used a model of METH self-administration (SA) that includes a punishment phase (footshocks) that helps to separate rats into a compulsive METH phenotype (compulsive) that continues to take METH and a non-compulsive METH (abstinent) group that suppressed or stopped taking METH. Animals were euthanized 2 h after the last METH SA session and their hippocampi were used to measure mRNA levels of cannabinoid receptors (*CB/Cnr*), as well as those of synthesizing (DAGL-A, DAGL-B, NAPEPLD) and metabolizing (MGLL, FAAH, PTGS2) enzymes of the endocannabinoid cascade. Non-compulsive rats exhibited significant increased hippocampal expression of *CB1/Cnr1* and *CB2/Cnr2* mRNAs. mRNA levels of the synthesizing enzyme, DAGL-A, and of the metabolic enzymes, MGLL and FAAH, were also increased. Non-compulsive rats also exhibited a significant decrease in hippocampal *Ptgs2* mRNA levels. Taken together, these observations implicate the hippocampal endocannabinoid system in the suppression of METH intake in the presence of adverse consequences.

**Supplementary Information:**

The online version contains supplementary material available at 10.1007/s12035-021-02656-8.

## Introduction

Methamphetamine (METH) is the most commonly used amphetamine-type stimulant (ATS) worldwide. About 1.6 million American adults aged 18 years or older had reported METH use between 2015 and 2018 [1]. Among these users, about 53% were reported to meet criteria for a METH use disorder (MUD) [1]. The acute behavioral effects of the drug are thought to be related to the fact that METH administration induces sustained dopamine (DA) release in the synaptic cleft [2, 3], with subsequent interactions with DA receptors [4, 5] that are located in various regions of the brain [6]. Importantly, repeated injections of METH can produce reactive oxygen species (ROS) [7, 8] that can alter the integrity of the brain structures [9] that are important for cognitive functions including learning and memory [10].

In addition to the effects on dopaminergic systems, a potential role of the endocannabinoid system (ECS) in the behavioral manifestations of rewarding drugs has been proposed [11–13]. The ECS is a bioactive lipid-based signaling pathway that includes cannabinoid receptors (CB1 and CB2) and the endogenous cannabinoids (eCBs), 2-arachidonoylglycerol (2-AG) and arachidonyl ethanolamide (AEA, anandamide) [14–16]. This system also includes synthesizing and degrading enzymes [17–21]. The eCBs are synthesized “on demand” by the enzymes diacylglycerol lipases (DAGL-A and DAGL-B) and N-acyl phosphotidylethanolamine phospholipase-D (NAPEPLD) that mediate the synthesis of 2-AG and AEA, respectively [19, 20]. 2-AG and AEA are, in turn, broken down by metabolic enzymes that are monoglyceride lipase (MGLL), fatty acid amide hydrolase (FAAH), and prostaglandin-endoperoxide synthase 2 (PTGS2) [17, 18, 21]. Both 2-AG and AEA bind to cannabinoid receptors (CB/Cnr) to facilitate downstream molecular changes [22].

Accumulating evidence suggests that eCBs (2-AG and AEA) modulate long-term synaptic plasticity in various brain regions [23]. However, the brain concentration of 2-AG is 170-fold higher than that of AEA [24], suggesting that 2-AG might play a primary role in the functions of that system in the brain. In addition, 2-AG binds preferentially to CB1 receptors in the hippocampus. CB1 receptors are expressed in GABAergic interneurons and glutamatergic axon terminals (reviewed by Kruk-Slomka et al. [14]) and modulate the maintenance of homeostatic eCB signaling [25] that is known to influence drug reward in various models [26, 27]. Owing to their localization, CB1 receptors participate in memory functions, stress, fear, and anxiety [28–30] by regulating neuronal signaling and synaptic plasticity [31]. Hippocampal eCB signaling is dependent on duration of the ligand in the synapse and ligand-CB1 binding efficacy. Importantly, the levels of eCBs in the brain are controlled by enzymatic degradation [32–36].

The discussion above thus indicated that the ECS signaling cascade could be either stimulated or inhibited in order to alter METH-induced neuroadaptation in preclinical models and as potential therapeutic approaches to counter MUD in humans. We were therefore interested in identifying potential effects of METH self-administration (SA) on ECS expression in the rats that can be consistently separated into compulsive and non-compulsive METH takers after the application of footshocks during METH SA [37–40]

## Materials and Methods

### Animals and Drug Treatment

We used 350–400 g male Sprague–Dawley rats from Charles River Labs, Raleigh, NC, USA, that were maintained in a temperature and humidity-controlled room (22.2 ± 0.2 °C) with sufficient access to food and water. The National Institute of Drug Abuse Animal Care and Use Committee approved our procedures that followed the Guide for the Care and Use of Laboratory Animals (ISBN 0–309–05,377–3).

### Intravenous Surgery

Using ketamine and xylazine (100 and 5 mg/kg, i.p., respectively), we anesthetized rats and placed silastic catheters into the jugular veins [38]. After surgery, the rats were monitored for health daily and the catheters were flushed every other day with sterile saline containing gentamicin (5 mg/ml; Butler Schein) and allowed to recover for 5–10 days before METH SA training. Rats received meloxicam (1 mg/kg, sc.) for analgesia upon awake from anesthesia and a second dose the following day.

### Training and Punishment Phases

METH self-administration training procedure is as previously described [38, 41]. Self-administration training was conducted in operant chambers equipped with two response levers—the “active” lever activates the infusion pump while inactive lever presses had no such program. In all the chambers, the rats were free to consume food and water from the feeders and water bottles hanging on the walls. Each press on the active lever will result in an infusion of dl-METH HCl (0.1 mg/kg/infusion) delivered at a volume of 0.1 ml during a 2–3-s period, accompanied by a 5-s compound tone-light stimulus. We used a fixed-ratio-1 schedule with 20-s timeout period designed to prevent drug overdose. The SA session lasted for 9 h/day (three 3-h sessions/day, each separated by 30 min off intervals) for 21 days. At the end of each 3-h session, the house light was turned off, and the active lever was retracted. For all rats, lever presses on the inactive lever are recorded but have no programmed consequences. In order to minimize weight loss, rats were trained in sets of 5 days of METH SA with 2 days off. During off days, rats were housed in the SA chambers disconnected from the intravenous SA lines.

The training conditions for the drug-naïve rats (controls, CT) will be the same as the METH-trained rats except sterile saline is infused after the press of the “active” lever.

Utilizing identical conditions as described above, rats continued METH SA during the punishment phase. Additionally, 50% of the reinforced lever presses for METH resulted in a simultaneous delivery of a 0.5-s footshock through the grid floor. The footshock currents were set to 0.18 mA on day 1, 0.24 mA on day 2, 0.30 mA on days 3 to 5, and 0.36 mA on days 6 to 8. The application of this shock intensity range has been shown to separate rats into shock-sensitive (SS) and shock-resistant (SR) animals [38, 42]. Furthermore, as a control for the effects of shock on biochemical and molecular markers within the brain, some saline rats will be yoked to the METH-trained rats that receive contingent shocks. The saline rats will also receive a footshock each time METH-trained animals received a contingent shock. There were separate groups of rats that were yoked to the corresponding shock-resistant (YSR) and shock-sensitive (YSS) rats, respectively.

### RNA Extraction

Using a guillotine 2 h after the last day of SA and footshocks, we euthanized the rats by decapitation and isolated hippocampus, nucleus accumbens (NAc), and mid-brain from the brains. Using Qiagen RNeasy Mini kit (Qiagen, Valencia, CA, USA), we were able to extract total RNA from individual brain samples of all the five groups (CT; SR; SS; YSR; YSS) and assessed its integrity with an Agilent 2100 Bioanalyzer (Agilent, Palo Alto, CA, USA); RNA samples showed no degradation and the RNA integrity numbers were > 8.0.

### Quantitative RT-PCR Analysis of mRNA Levels

Using Advantage RT-for-PCR kit (Clontech, Mountain View, CA, USA), we reverse-transcribed 500 ng of total RNA from the CT group (*n* = 9), SR group (*n* = 8), SS group (*n* = 7), YSR group (*n* = 12), and YSS (*n* = 8) into cDNA. Quantitative polymerase chain reaction (PCR) was carried out as described by our published protocol [43]. The gene-specific primers were synthesized from the Synthesis and Sequencing Facility of Johns Hopkins University (Baltimore, MD, USA) based on the PCR primers we generated using LightCycler probe design software v. 2.0 (Roche Biosystems, Indianapolis, IN, USA). The list of primers used is given in Table S1. Quantitative reverse transcription PCR was performed using the Roche LightCycler 480 II with iQ SYBR Green Supermix (Bio-Rad). A standard curve method was used to determine the concentration of unknown samples. The raw data was obtained using a second derivative maximum analysis via a non-linear, polynomial regression line (Roche Light cycler software). Data reported uses absolute quantification. Within each sample, the relative amounts of mRNA analyzed were normalized using two reference genes *Clathrin* and *18S*. The results are shown as fold changes calculated as the ratios of normalized gene expression data for METH-treated groups (SR and SS) compared to its respective yoked shock groups (YSR and YSS) and to the CT control group.

### Statistical Analysis

Behavioral and RT-PCR data were analyzed using Prism® version 6 (San Diego, CA). Behavioral data were analyzed using repeated measures two-way ANOVA. The dependent variable was the total number of METH infusions for each group on each day. Independent variables were the within-subject factor group (SS or SR), between-subject factor training session duration, and their interactions. Bonferroni post hoc tests were used to compare METH intake between SS and SR groups. RT-PCR data were analyzed using one-way analysis of variance (ANOVA) followed by Fisher’s PLSD post hoc test. ANOVA was also used to analyze the total number of shocks received by the yoked shock groups. Statistical significance for all tests was set at *p* < 0.05.

## Results

### Effects of Footshocks on METH Infusion

**Fig. 1:**
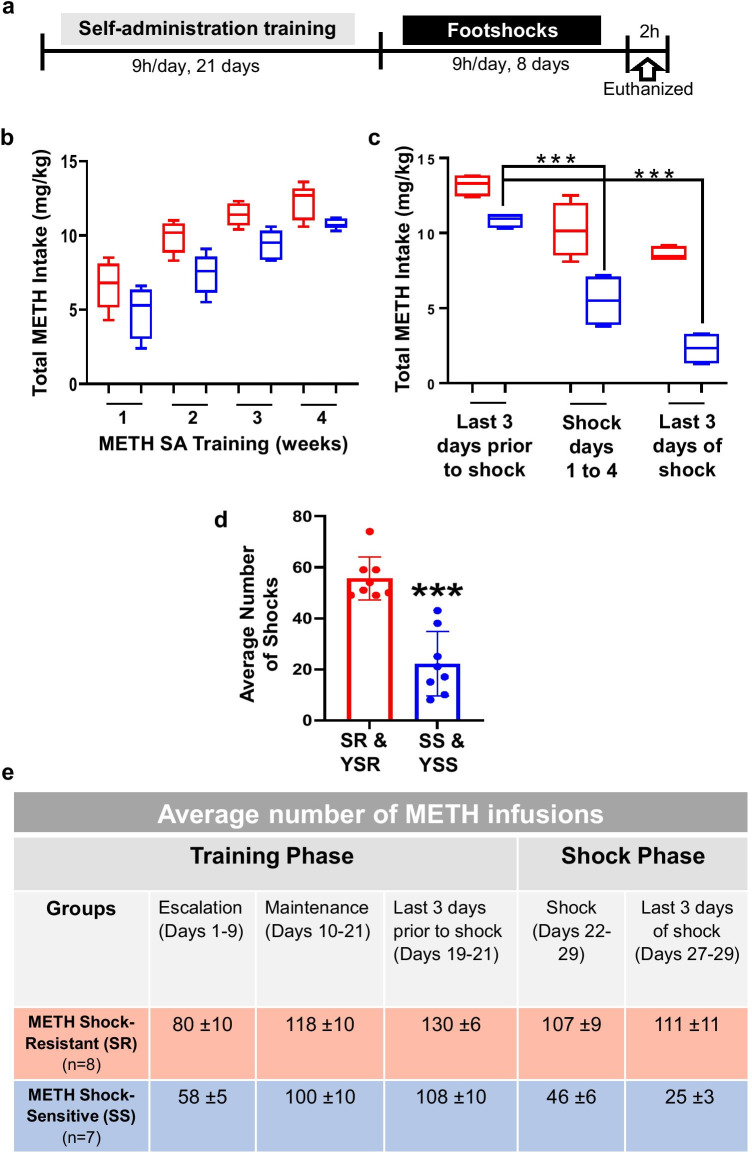
The behavioral effect of contingent footshock punishment on METH intake. The experimental timeline is given (a), where rats self-administered METH for 21 days followed by footshocks for 8 days. The drug-naïve control rats (CT) will be the same as the METH-trained rats except sterile saline is infused after the active lever press. The control animals for footshock also received saline but were yoked to the METH-trained rats that receive contingent shocks. There were separate groups of rats that were yoked to the corresponding METH shock-resistant (SR) and shock-sensitive (SS) rats. They are termed YSR and YSS, respectively. (b) Box and whisker plot shows that during weeks 1 and 2 rats given access to METH slowly increased their drug intake (*escalation phase*). By weeks 3 and 4, the METH intake plateaued (*maintenance phase*). Total METH intake between shock-resistant (SR, *n* = 8, shown as red) and shock-sensitive (SS, *n* = 7, shown as blue) rats did not significantly differ during these *escalation* and *maintenance* phases. (c) Following the footshock punishment, SS rats significantly decreased their total METH intake (*p* < 0.0001), while the SR rats did not. (d) The column bar graph shows the number of footshocks received by the animals. The SS and yoked shock-sensitive (YSS) rats received a significantly lower number of footshocks compared to the SR and yoked shock-resistant (YSR) rats (*p* < 0.001). (e) The table depicts the average number of METH infusions consumed by rats during both the training and punishment phases

As reported in Subu et al. [40], rats were first trained for 21 days of METH SA (SA training phase) prior to the application of 8 days of contingent footshocks. The experimental paradigm is shown in Fig. 1a. Contingent footshocks separated METH SA rats into shock-sensitive (SS, non-compulsive, *n* = 7) and shock-resistant (SR, compulsive, *n* = 8) groups. SS rats significantly decreased their METH intake during the shock phase whereas the SR rats continued to compulsively press the lever to obtain METH. The control (CT, *n* = 9) and rats yoked to SR and SS during the footshock regimen (YSR—yoked shock resistant, *n* = 12; YSS—yoked shock sensitive, *n* = 8) self-administered saline solution throughout the experiment. The box and whisker plots in Fig. 1b show that there were no significant differences in total METH intake between SR and SS rats during the escalation (weeks 1 and 2; SR: 8.0 ± 0.1, SS: 5.8 ± 0.05) and maintenance phases (weeks 3 and 4; SR: 11.8 ± 0.1, SS: 10.0 ± 0.1) of METH SA. In contrast, following footshocks, SS rats self-administered significantly lower amounts of METH than SR rats (Fig. 1c). Total METH intake for SR rats on the last 3 days prior to shock (13.5 ± 0.8) did not significantly differ from the last 3 days of shock (11.1 ± 0.11). However, significant decreases in total METH intake were observed for SS rats, with intake over the last 3 days of shock (2.5 ± 0.3) being markedly lower than the 3 days prior to shock (10.8 ± 0.10) (see Fig. 1c). Figure 1d shows that there was a lower number of footshocks received by the SS and YSS rats in comparison to the SR and YSR rats (*p* < 0.001). Figure 1e illustrates the actual number of METH infusions taken by the rats during both the training and shock phases of the behavioral experiment.

### Effects of METH SA and Contingent Footshock on mRNA Expression of Cannabinoid Receptors (CB/Cnr) in the Rat Hippocampus

**Fig. 2:**
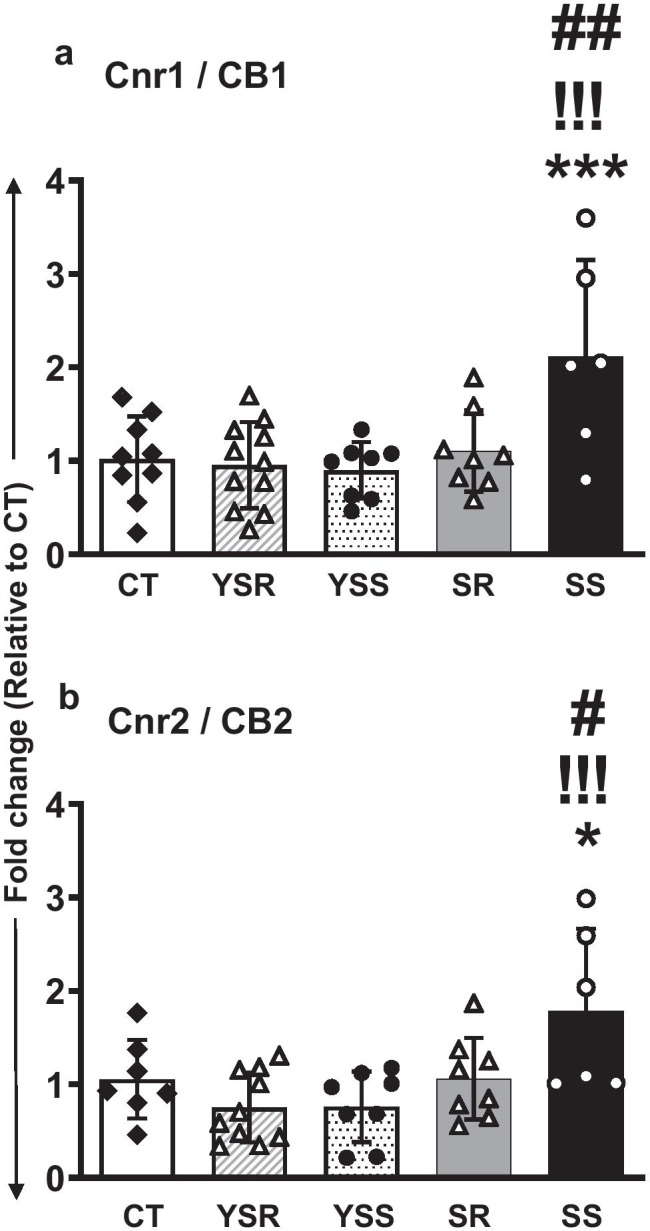
Altered rat hippocampal mRNA expression of cannabinoid receptors (CB/Cnr) as a result of METH SA followed by contingent footshocks. Quantitative RT-PCR (qRT-PCR) data on the mRNA expression of the cannabinoid receptors, *CB1/Cnr1* (a) and *CB2/Cnr2* (b). One-way ANOVA analysis of the data revealed significant changes in CB1/Cnr1 expression (*p* = 0.0001) and CB2/Cnr2 expression (*p* = 0.0017). Post hoc analyses showed significant increases in both receptor mRNA levels in the SS group compared to the CT, YSS, and SR groups. Key to statistics: **P < 0.01, ***P < 0.001, compared with controls; !!!*P* < 0.001, comparison to yoked shock sensitive (YSS); #*P* < 0.05, ###*P* < 0.001, comparison to compulsive METH (SR) group

Results from quantitative RT-PCR (qRT-PCR) that measured the mRNA expression of the cannabinoid receptors—*CB1/Cnr1* and *CB2/Cnr2*—are shown in Fig. 2a and b, respectively. One-way ANOVA analysis of the qRT-PCR data revealed significant changes in the expression of *CB1/Cnr1* [*F*_(4,37)_ = 5.618, *p* = 0.0012] and *CB2/Cnr2* [*F*_(4,34)_ = 4.801, *p* = 0.003]. Post hoc analyses revealed that these changes were due to significant increases in *CB1/Cnr1* and *CB2/Cnr2* mRNA levels in the SS rats in comparison to CT, YSS, and SR rats (Fig. 2b and c).

### Effects of METH SA and Contingent Footshock on mRNA Expression of Cannabinoid Enzymes in the Rat Hippocampus

**Fig. 3:**
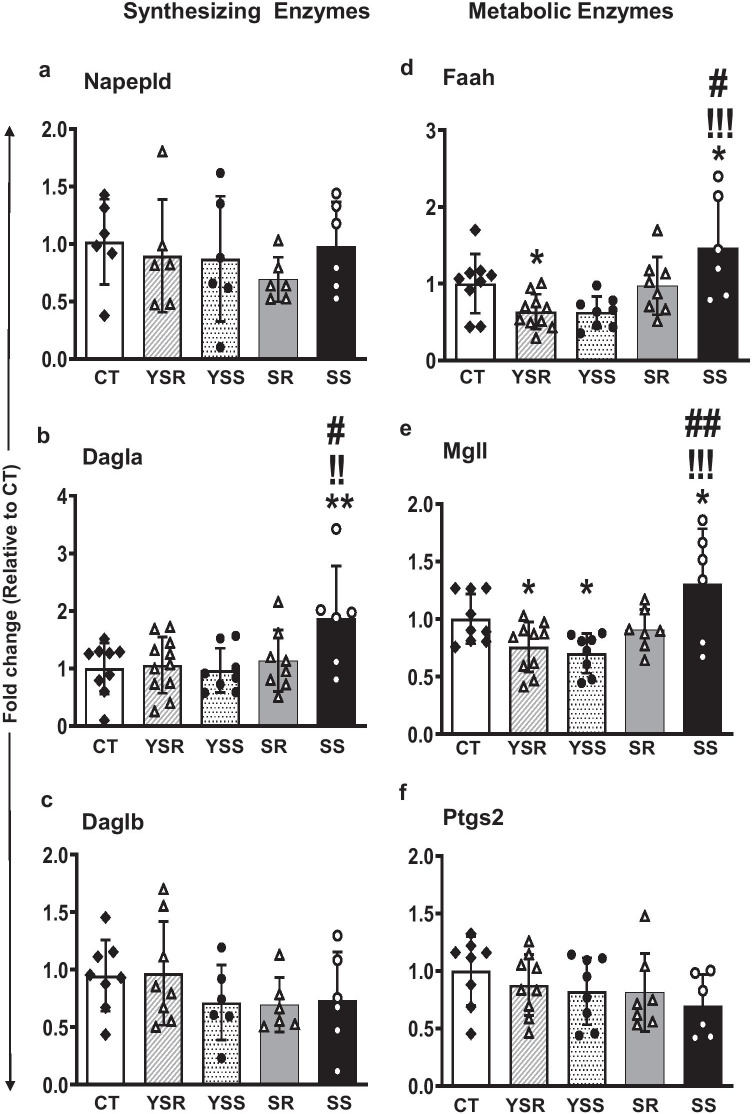
Effect of METH SA and contingent footshock on rat hippocampal mRNA expression of key cannabinoid enzymes within the ECS cascade. Data from qRT-PCR showed significant increase in the mRNA expression of the synthesizing enzyme *Dagla* (b) in SS rats in comparison to CT, YSS, and SR rats (*p* < 0.01). Among the metabolizing enzymes, significant changes in the expression of Faah (*p* = 0.0004) and Mgll (*p* = 0.0015) were also observed where significant increases in mRNA levels were noted in the SS rats compared to the YSS, YSR, and SR rats. Significant decreases in Ptgs2 (*p* = 0.0003) mRNA levels were noted in the SS, YSR, and YSS groups in comparison to the CT group. Key to statistics: *P < 0.05, **P < 0.01, compared with controls; !!!*P* < 0.001, comparison to yoked shock sensitive (YSS); #*P* < 0.05, ##*P* < 0.01, comparison to compulsive METH (SR) group

The effects of METH SA and footshock on the five key synthesizing enzymes that participate in the ECS signaling cascade systems are shown in Fig. 3. Among the synthesizing enzymes, we found significant changes in *Dagla* [*F*_(4,37)_ = 3.042, *p* = 0.029] mRNA expression (Fig. 3b), with post hoc tests identifying increased *Dagla* expression in SS rats in comparison to the CT, YSS, and SR rats. No significant changes were observed in *Napepld* (Fig. 3a) and *Daglb* (Fig. 3c) mRNA expression.

The METH SA-induced alterations in mRNA levels of metabolizing enzymes of the ECS pathway identified significant changes in the expression of *Faah* [*F*_(4,36)_ = 5.857, *p* = 0.001] (Fig. 3d) and *Mgll* [*F*_(4,35)_ = 6.015, *p* = 0.0009] (Fig. 3e). Post hoc analyses revealed significant increases in mRNA levels in the SS rats in comparison to YSS and SR rats. No significant changes was seen in the expression of *Ptgs2* [*F*_(4,33)_ = 0.976, *p* = 0.4333] (Fig. 3f).

## Discussion

The present study documents, for the first time, alterations in the expression of eCB genes in compulsive METH taking and non-compulsive rats divided after application of footshocks after all the rats had escalated their METH intake. Non-compulsive METH takers showed increased hippocampal mRNA expression of both cannabinoid receptors—*CB1/Cnr1* and *CB2/Cnr2*—but exhibited no changes in the NAc and mid-brain (supplementary figure S1). They also exhibited increased hippocampal expression of *Dagla*, *Mgll*, and *Faah* enzymes but no alterations in the NAc and mid-brain (supplementary figure S2). Together, these observations support the idea that the hippocampal ECS signaling cascade may be specifically involved in some of the behavioral manifestations of METH SA in animals and, by extension, METH use disorder (MUD) in humans [44–46].

Our findings of increased expression of hippocampal metabolizing enzymes, *Mgll* and *Faah*, are consistent, in part, with those of Blanco et al. [47] who had reported an increase in gene and protein expression of MGLL and FAAH in the hippocampus following both acute (10 mg/kg) and repeated (20 mg/kg) cocaine administration for five consecutive days. These authors also reported increased *CB1/Cnr1* expression after cocaine [47]. Nevertheless, we found more changes in the non-compulsive (SS) rats that had significantly decreased their intake after punishment. These observations suggest that eCB signaling may be influenced by the differences in neuronal activity related to the quantities of METH taken by the two divergent groups. On the other hand, the changes might be related to the ability of some rats to learn the association of lever pressing for METH with the application of footshocks. In fact, although all the rats had learned to self-administer METH during the initial training phase, some of the rats seem to be more apt to learn the association of punishment with lever pressing for METH. These rats reduce their lever pressing activities whereas others might not have learned the association and continued to lever press despite the adverse consequences. This suggestion is consistent with the work of Marsicano et al. [48] who showed that blockade of CB1 receptor with the antagonist, SR141716, led to impaired extinction training after aversive stimulus. The discussion is also supported by Shiflett et al. [49] who also reported that intra-hippocampal infusion of SR141716 leads to increased memory but reduced flexibility and adaptation to new environmental conditions.

Our results suggest that exposure to METH can impact the hippocampal ECS. For example, stimulus-induced post-synaptic eCB formation is important in inducing neurogenesis [50, 51]. In addition, the participation of that system in the promotion of neurogenesis is supported by observations that cannabinoid receptor *CB1/Cnr1*-deficient animals showed decreased whereas FAAH-deficient ones showed increased proliferation of neural progenitor cells [52, 53]. Interestingly, decreased neurogenesis in the hippocampus of rodents has been reported after exposure to METH SA [54]. Our findings of increased CB receptor expression suggest that there might be increased neurogenesis in animals that decreased their METH intake in the presence of footshocks since CB1-deficient rodents showed decreased neurogenesis [52, 53]. This discussion is consistent with the results of Galinato et al. [55] that indicate the presence of increased neurogenesis during withdrawal from METH SA.

It is also possible that the increased CB mRNA levels in the non-compulsive rats might have served to suppress METH taking behaviors via inhibition of the release of glutamate [56, 57] which plays a role in drug SA [58–61]. The possibility that the increased expression of CB receptors might be due to compensatory increases secondary to decreased levels of eCBs because of METH-induced increases in their metabolism needs to be taken into consideration. This suggestion stems from our observations of increased expression of the metabolizing enzymes, *Mgll* and *Faah*, in the hippocampus of the non-compulsive rats. This supposition is consistent with the observations of Bystrowska et al. [62] who showed that exposure to cocaine SA for 14 days decreased hippocampal 2-AG, the MGLL substrate.

In contrast to our findings, URB597, an inhibitor of FAAH, was reported to suppress METH-seeking behavior in mice [63]. The dichotomous findings may be related to species differences since FAAH inhibition was found to increase nicotine [64] and alcohol reward [65] in mice, but reduced nicotine [66] or alcohol [67] reward in rats.

Our findings of increased hippocampal *Dagla* expression in the non-compulsive rats are consistent, in part, with the results of Mitra et al. [68] who reported increased levels of DAGL after prolonged abstinence (30 days) from cocaine SA. Together, these results suggest that abstinence for SA of psychostimulants may be accompanied by increased synthesis of 2-AG that might lead to increased synthesis of metabolizing enzymes in order to return the system back to homeostasis. These suggestions will need to be more thoroughly investigated by measuring the eCB levels in both the phenotypes.

**Fig. 4:**
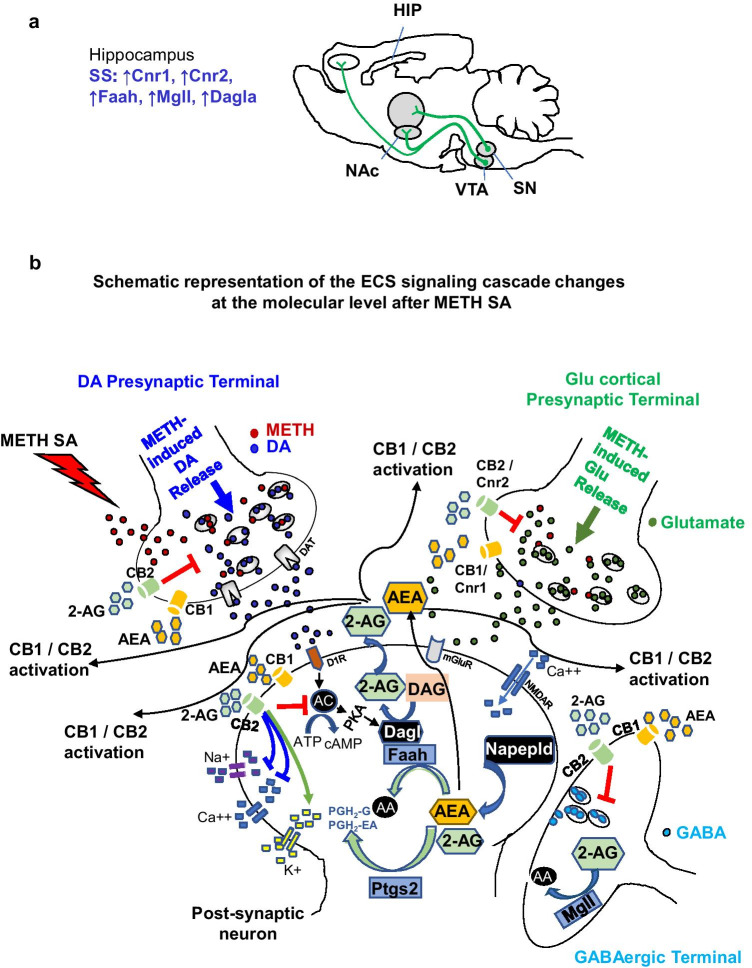
Schematic representation of a potential mechanism within the rat hippocampal ECS signaling cascade as a result of METH SA. (a) The rat brain is depicted with specific changes noted within the hippocampi of SS rats—increased levels of *Cnr1*, *Cnr2*, *Faah*, and *Mgll*. (b) The schema helps to better understand the effect of the training and shock phases. For instance, SS rats had elevated levels of *Cnr1* and *Cnr2* suggesting an upregulation of the receptor in order to combat the neurotoxic effects of dysregulated dopamine/glutamate release due to METH intake. As a result, 2-AG and AEA, produced by DAGL and NAPEPLD, would increase and bind to these receptors to prevent excess release of these neurotransmitters. FAAH and MGLL then work to break down the excess endocannabinoids to prevent unnecessary activation of the cannabinoid receptors. Abbreviations: AA arachidonic acid, AC adenylate cyclase, PGH prostaglandin

In conclusion, we show, for the first time, that the endogenous ECS is significantly impacted in rats that had suppressed their METH intake in the presence of footshocks. Figure 4 shows a schematic illustration of the potential impact of METH SA on the ECS signaling cascade in the hippocampus. Future research is necessary to elucidate the potential role of the ECS in METH-induced changes in neurogenesis, oxidative stress, and neurodegenerative processes in various brain regions.

## Supplementary Information

Below is the link to the electronic supplementary material.

Supplementary file1 (DOCX 12 KB)

Supplementary file2 (DOCX 15 KB)

## Data Availability

All data generated and analyzed are included in this article.

## Abbreviations

2-AG: 2-Arachidonoylglycerol
AEA: Anandamide
CB/Cnr: Cannabinoid receptor
CT: Control group
DAGL: Diacylglycerol lipase
eCB: Endocannabinoid
ECS: Endocannabinoid system
FAAH: Fatty acid amide hydrolase
METH: Methamphetamine
MGLL: Monoglyceride lipase
NAPEPLD: N-acyl phosphatidylethanolamine phospholipase-D
NAc: Nucleus accumbens
PTGS2: Prostaglandin-endoperoxide synthase 2
SA: Self-administration
SR: Shock-resistant group
SS: Shock-sensitive group
THC: Tetrahydrocannabinol
YSR: Yoke shock resistant
YSS: Yoke shock sensitive
