# Supplementary material for: Footshock-Induced Abstinence from Compulsive Methamphetamine Self-administration in Rat Model Is Accompanied by Increased Hippocampal Expression of Cannabinoid Receptors (CB1 and CB2)
Source: Mol Neurobiol. 2022 Jan 3;59(2):1238–48. doi: 10.1007/s12035-021-02656-8 (PMC8857101; doi:10.1007/s12035-021-02656-8)
Supplement: Supplementary file 1 — Supplementary file1 (DOCX 12 KB) [file 12035_2021_2656_MOESM1_ESM.docx]

**Figure legends for supplementary figures**

**Figure S1. mRNA expression of cannabinoid receptors (CB/Cnr) in the nucleus accumbens (a and b), and midbrain (c).** One-way ANOVA followed by post-hoc analyses of the data revealed no significant changes in CB1/Cnr1 expression between the two phenotypes (shock-resistant, SR and shock-sensitive, SS) in both nucleus accumbens (a) and mid-brain. (c). Similarly, no changes and in CB2/Cnr2 expression was observed in the nucleus accumbens (b). CB2/Cnr2 mRNA expression was not detected in the midbrain.

**Figure S2. Effect of METH SA and contingent footshock on mRNA expression of key cannabinoid enzymes within the ECS cascade in the nucleus accumbens (a – c; g – i) and midbrain (d – f; j – l).** One-way ANOVA followed by post-hoc analyses of the data revealed no significant changes in the mRNA expression of ECS cascade enzymes between the two phenotypes (shock-resistant, SR and shock-sensitive, SS). Key to statistics: **P < 0.01, compared with controls.
