# Supplementary material for: Footshock-Induced Abstinence from Compulsive Methamphetamine Self-administration in Rat Model Is Accompanied by Increased Hippocampal Expression of Cannabinoid Receptors (CB1 and CB2)
Source: Mol Neurobiol. 2022 Jan 3;59(2):1238–48. doi: 10.1007/s12035-021-02656-8 (PMC8857101; doi:10.1007/s12035-021-02656-8)
Supplement: Supplementary file 2 — Supplementary file2 (DOCX 15 KB) [file 12035_2021_2656_MOESM2_ESM.docx]

**Table S1.** **List of RT-PCR primers sequences**

| **Gene Name** | **Forward** | **Reverse** |
| --- | --- | --- |
| *CB1 / Cnr1* | GGC ATC CAA ATT AGG ATA CT | CGA CGA GAG AGA CTT GTT AT |
| *CB2 / Cnr2* | GTT AAC TCC ATG ATC AAT CCT A | CTC AGC CTC TGT CTC TGT AA |
| *Napepld* | TTA TGA GCC AAG GTG GTT TA | TAT TCA AGT ATC GTG ACT CTC C |
| *Dagla* | TCC TTC TGA GCT GTA TGA TT | TCC CGA GAG TGA CAT TCT TA |
| *Daglb* | TTG TTC TCC TGG CTG TTA TT | CTG TCA CAT TGG ATA CCT TT |
| *Faah* | CAG CAT GAG ATT GAG ATG TAT | CAA AGT AGC CTT TGT AGA GT |
| *Mgll* | TTT GTC CTG CCA AAC ATA TC | TAG GCA CCT TCA TAC ATC TT |
| *Ptgs2* | CCG GGT CTG ATG ATG TA | CTC AGG TGT TGC ACG TA |
